# Dual-morphology Bundle Branch Re-entrant Ventricular Tachycardia in Non-dilated Cardiomyopathy

**DOI:** 10.19102/icrm.2025.16045

**Published:** 2025-04-15

**Authors:** Ibrahim Alshaghdali, Tyler Alderson, Hakan Paydak, John Paul Mounsey, Subodh Devabhaktuni

**Affiliations:** 1 University of Arkansas for Medical Sciences, Little Rock, AR, USA; 2 University of Ha’il College of Medicine, Ha’il, Saudi Arabia; 3 Division of Cardiovascular Disease, University of Missouri Hospitals, Columbia, MO, USA; 4 Division of Cardiology, Department of Medicine, University of Arkansas for Medical Sciences, Little Rock, AR, USA

**Keywords:** Bundle branch re-entry, catheter ablation, ventricular tachycardia

## Abstract

Bundle branch re-entrant (BBR) tachycardia is an uncommon form of ventricular tachycardia (VT). This arrhythmia typically occurs in patients with a structural heart disease, especially dilated cardiomyopathy, and significant conduction system impairment, although affected patients with a structurally normal heart or normal conduction system have been reported. The QRS morphology during tachycardia can vary; it typically has a left bundle branch block (LBBB) morphology in which the antegrade conduction is over the right bundle and the retrograde limb is over the left bundle. The reverse of this circuit results in a right bundle branch block (RBBB) QRS morphology. A re-entrant circuit also can utilize interfascicular conduction, such as antegrade conduction over the left anterior fascicle and retrograde conduction up the left posterior fascicle or vice versa. Although there are reports of BBR tachycardia and interfascicular VT occurring in the same patient, to our knowledge, there are no prior reports of BBR tachycardia that has both LBBB and RBBB morphologies in the same patient. This case illustrated a BBR tachycardia with both left bundle and right bundle branch morphologies occurring in a patient with a non-dilated left ventricle.

## Introduction

Bundle branch re-entrant (BBR) tachycardia is an underrecognized form of ventricular tachycardia (VT). This arrhythmia typically occurs in patients with a structural heart disease, especially dilated cardiomyopathy, and significant conduction system impairment, although affected patients with a structurally normal heart or normal conduction system have been reported^1,2^.

The QRS morphology during tachycardia can vary. It typically has a left bundle branch block (LBBB) morphology, in which the antegrade conduction is over the right bundle and the retrograde limb is over the left bundle. The reverse of this circuit results in a right bundle branch (RBB) block (RBBB) QRS morphology. A re-entrant circuit also can use interfascicular conduction, such as antegrade conduction over the left anterior fascicle and retrograde conduction up the left posterior fascicle^3^ or vice versa.

Although there are reports of the BBR tachycardia and interfascicular VT occurring in the same patient^4^, we found no prior reports of BBR tachycardia that has both LBBB and RBBB morphologies in the same patient.

## Case presentation

**Figure 1:**
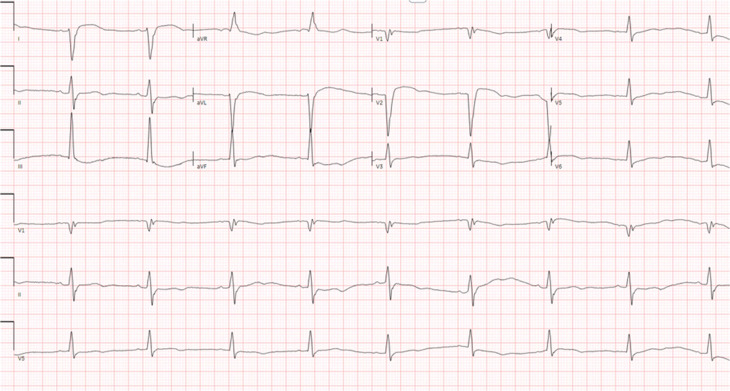
Baseline electrocardiogram.

**Figure 2:**
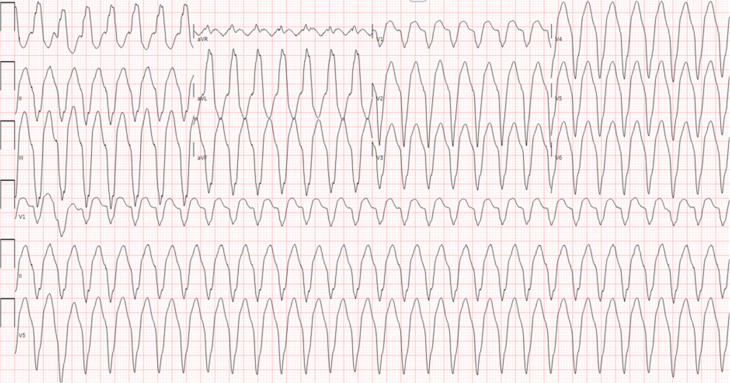
Pre-procedure wide complex tachycardia.

This is a case of a 38-year-old woman with a prior medical history of symptomatic tachycardia, type II diabetes mellitus, hypertension, and polycystic ovarian syndrome. She was seen by a cardiologist out of state for her tachycardia and was treated with sotalol for 3 years. She does not have a history of a prior ablation. She then presented to an outpatient electrophysiology (EP) clinic to establish care. She reported recent recurrent episodes of palpitations with associated jaw pain lasting for >7 h. Her baseline electrocardiogram (ECG) is shown in **Figure 1**, which shows sinus bradycardia with a left posterior fascicular block (LPFB). An ECG taken during one of the episodes is shown in **Figure 2**. A transthoracic echocardiogram was obtained, which revealed mildly increased left ventricular wall thickness, normal left ventricular internal dimensions, and mildly reduced left ventricular systolic function with an estimated left ventricular ejection fraction of 40%. The left ventricle showed diffuse global hypokinesis with no wall motion abnormalities. Subsequently, cardiac magnetic resonance imaging was obtained, which showed mild diffuse global hypokinesis with areas of dyskinetic segments in the interventricular septum. A reduced left ventricular ejection fraction of 37% with normal left ventricular dimensions and mild diffuse left ventricular wall thickening were observed. Focal mid-myocardial enhancement in the inferolateral wall at the base and mid-ventricular level is suggestive of prior myocarditis and scar.

**Figure 3:**
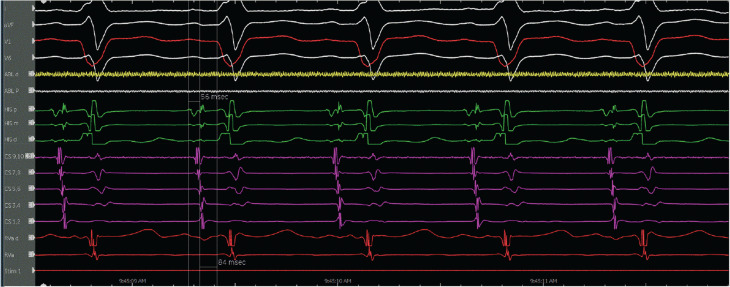
A–H to H–V interval.

**Figure 4:**
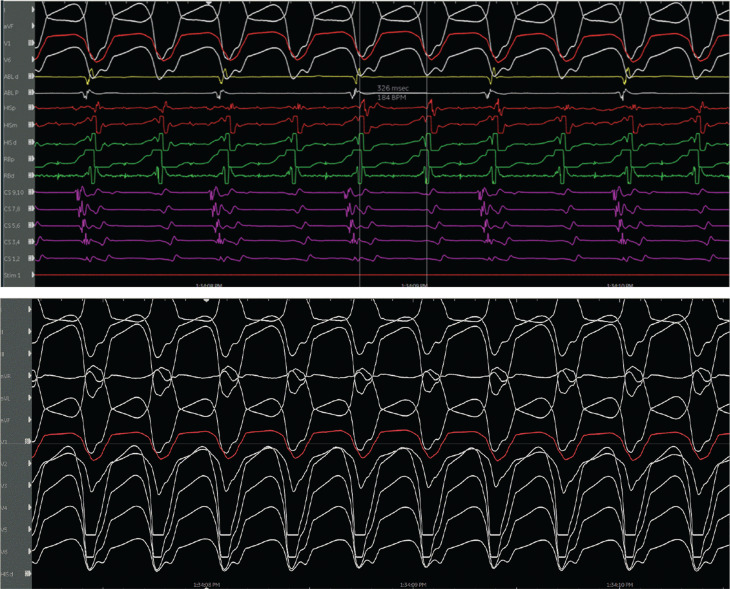
Ventricular tachycardia with a left bundle branch block morphology and a 12-lead electrocardiogram of the tachycardia.

**Figure 5:**
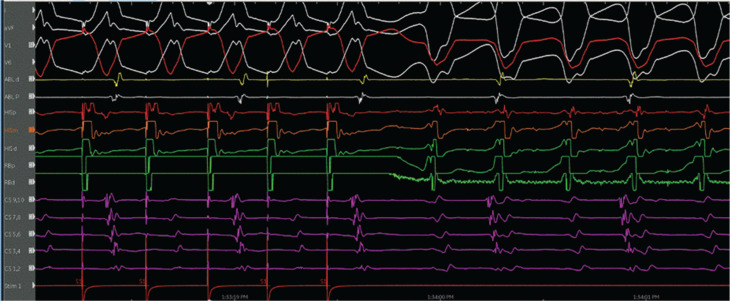
Initiation of ventricular tachycardia with a left bundle branch block morphology by burst atrial pacing.

**Figure 6:**
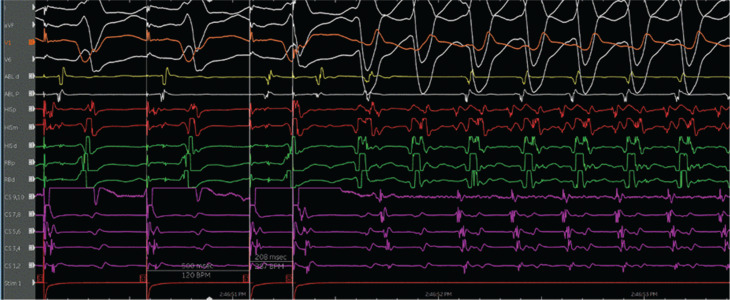
Initiation of ventricular tachycardia with a right bundle branch block morphology with single extra atrial stimulation.

**Figure 7:**
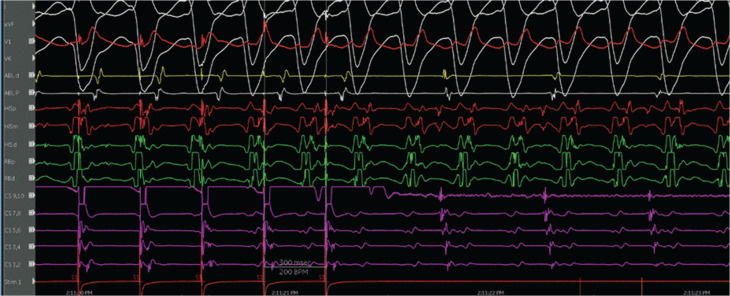
Initiation of ventricular tachycardia with a right bundle branch block morphology with decremental atrial pacing.

**Figure 8:**
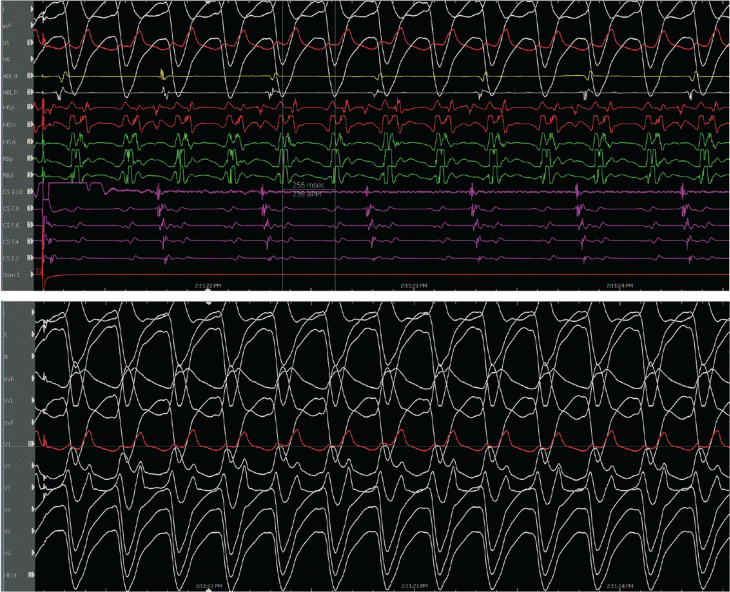
Ventricular tachycardia with a right bundle branch block morphology and a 12-lead electrocardiogram of the tachycardia.

The patient was referred for an EP study with possible ablation. Her baseline ECG during the EP study was notable for sinus rhythm with an LPFB. She had an A–H interval of 56 ms and an H–V interval of 84 ms **(Figure 3)**. VT was initiated with atrial pacing from the proximal coronary sinus (CS) catheter (CS site 6), as detailed later, on isoproterenol up to 10 μg/min. At first, VT with a typical LBBB morphology with a cycle length of 279 ms was induced by burst pacing **(Figures 4 and 5)**. It terminated spontaneously. Using a single atrial extra stimulus **(Figure 6)** and decremental atrial pacing **(Figure 7)**, from the same site, a tachycardia with a typical RBBB morphology **(Figure 8)** was induced with a cycle length of 250 ms.

**Figure 9:**
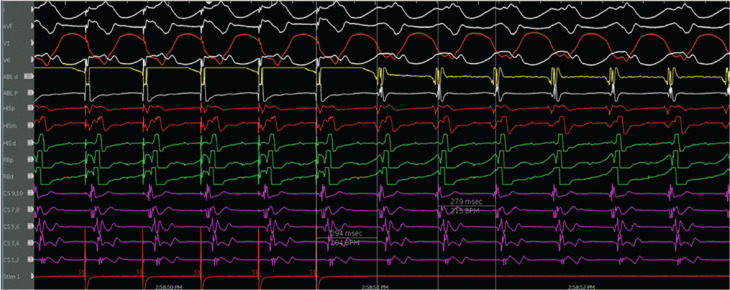
Post-pacing interval–tachycardia cycle length (PPI–TCL) during tachycardia and right ventricular apical pacing. The PPI–TCL (294–279 ms) with overdrive apical right ventricular pacing was 15 ms, suggestive of a site with proximity to the circuit. This electrogram also shows the activation pattern of the His bundle and right bundle during tachycardia.

**Figure 10:**
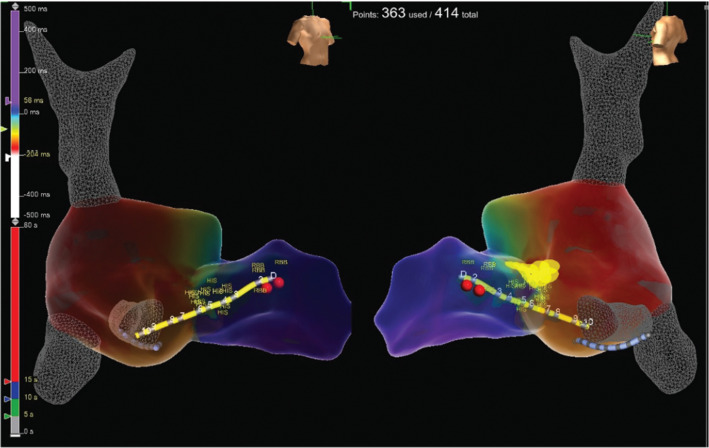
Site of ablation represented by red circles on a three-dimensional map.

**Figure 11:**
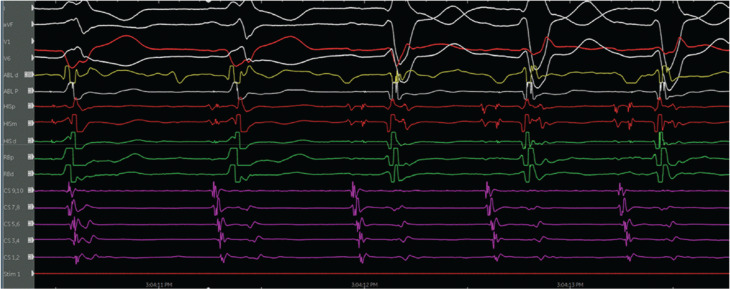
Change to right bundle branch block during ablation.

**Figure 12:**
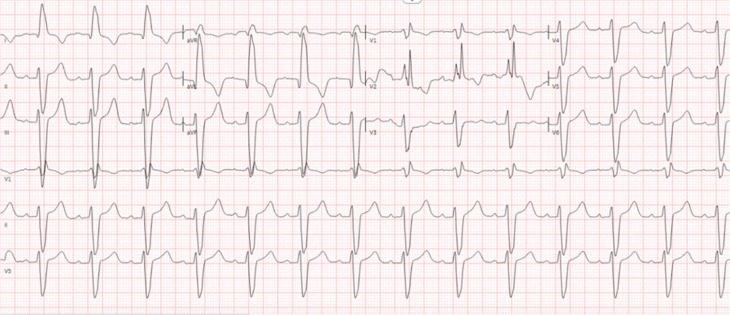
Post-procedure electrocardiogram.

The evidence for the presence of a macro–re-entrant mechanism for both VTs includes the reproducible inducibility of the tachycardias using programmed stimulation and the ability to entrain the tachycardia from the multiple sites (surface ECG fusion demonstrated). The evidence for the presence of a BBR tachycardia included a His potential preceding each ventricular activation with an H–V interval greater than that recorded during sinus rhythm. Spontaneous changes in the H–H intervals preceded similar changes in the V–V intervals. The post-pacing interval–tachycardia cycle length (294–279 ms) with overdrive apical right ventricular pacing was 15 ms, suggestive of a site with proximity to the circuit **(Figure 9)**. A 2:1 ventriculo-atrial conduction during tachycardia excluded an atrioventricular re-entrant tachycardia. Furthermore, His activation during tachycardia was noted to be distal to proximal, and RB activation during tachycardia was proximal to distal. Radiofrequency catheter ablation during sinus rhythm at the RBB site **(Figure 10)** (presence of RBB potential on ablation catheter at distal bipole with no local atrial electrogram) at an energy of 20–35 W was performed for 60 s. An RBBB developed after 32 s **(Figures 11 and 12)**. A secondary application just proximal to the prior ablation site was delivered for 30 s. After the ablation, VT was no longer inducible. No other morphologies of VT, either sustained or non-sustained, could be initiated. The H–V interval post-procedure remained unchanged at 84 ms.

Written procedural consent was obtained from the patient.

### Outcome and follow-up

The patient had no recurrence of VT after 4 months of follow-up. Her 3-month follow-up transthoracic echocardiogram showed an improvement in her ejection fraction to 50%.

## Conclusion

BBR tachycardia is an underrecognized form of VT. This case illustrated a BBR tachycardia with both left bundle branch and RBB morphologies occurring in a patient with a non-dilated left ventricle. There have been case reports of BBR and interfascicular VT occurring in the same patient; however, there are no reports of LBBB and RBBB QRS morphology VT occurring in the same patient, especially in non-dilated cardiomyopathy, to the best of our knowledge.
